# Osteoinduction and proliferation of bone-marrow stromal cells in three-dimensional poly (ε-caprolactone)/ hydroxyapatite/collagen scaffolds

**DOI:** 10.1186/s12967-015-0499-8

**Published:** 2015-05-08

**Authors:** Ting Wang, Xiaoyan Yang, Xin Qi, Chaoyin Jiang

**Affiliations:** Department of Orthopedics, Shanghai Jiao Tong University Affiliated Sixth People’s Hospital, No.600 Yishan Rd, Shanghai, 200233 China; Department of Medicine, Northwestern University, Chicago, IL 60208 USA

**Keywords:** Poly(ε-caprolactone), Hydroxyapatite and collagen coating, 3D culture

## Abstract

**Background:**

Osteoinduction and proliferation of bone-marrow stromal cells (BMSCs) in three-dimensional (3D) poly(ε-caprolactone) (PCL) scaffolds have not been studied throughly and are technically challenging. This study aimed to optimize nanocomposites of 3D PCL scaffolds to provide superior adhesion, proliferation and differentiation environment for BMSCs in this scenario.

**Methods:**

BMSCs were isolated and cultured in a novel 3D tissue culture poly(ε-caprolactone) (PCL) scaffold coated with poly-lysine, hydroxyapatite (HAp), collagen and HAp/collagen. Cell morphology was observed and BMSC biomarkers for osteogenesis, osteoblast differentiation and activation were analyzed.

**Results:**

Scanning Electron Microscope (SEM) micrographs showed that coating materials were uniformly deposited on the surface of PCL scaffolds and BMSCs grew and aggregated to form clusters during 3D culture. Both mRNA and protein levels of the key players of osteogenesis and osteoblast differentiation and activation, including runt-related transcription factor 2 (Runx2), alkaline phosphates (ALP), osterix, osteocalcin, and RANKL, were significantly higher in BMSCs seeded in PCL scaffolds coated with HAp or HAp/collagen than those seeded in uncoated PCL scaffolds, whereas the expression levels were not significantly different in collagen or poly-lysine coated PCL scaffolds. In addition, poly-lysine, collagen, HAp/collagen, and HAp coated PCL scaffolds had significantly more viable cells than uncoated PCL scaffolds, especially scaffolds with HAp/collagen and collagen-alone coatings. That BMSCs in HAp or HAp/collagen PCL scaffolds had remarkably higher ALP activities than those in collagen-coated alone or uncoated PCL scaffolds indicating higher osteogenic differentiation levels of BMSCs in HAp or HAp/collagen PCL scaffolds. Moreover, morphological changes of BMSCs after four-week of 3D culture confirmed that BMSCs successfully differentiated into osteoblast with spread-out phenotype in HAp/collagen coated PCL scaffolds.

**Conclusion:**

This study showed a proof of concept for preparing biomimetic 3D poly (ε-caprolactone)/ hydroxyapatite/collagen scaffolds with excellent osteoinduction and proliferation capacity for bone regeneration.

## Introduction

Bone consists mainly of collagen fibres and an inorganic bone mineral in the form of small crystals [1]. The main constituents of human bones are bone mineral (~60–70 wt%), collagen (~10–30 wt%) and water (~10-20 wt%) [2-5]. Most of the rest is collagen, and bone also contains a small amount of other proteins and inorganic salts [6]. As a renewable tissue, it can spontaneously repair its slight loss or minor damages by regenerating [7,8]. However, treatment of large segmental bone defects, which often involves bone repair or replacement, remains as an unsolved medical challenge for clinicians and researchers [9,10]. Bone diseases including osteogenesis imperfecta, osteoarthritis, osteomyelitis, paralleled with fractures and traumas, as well as tumor resection can all lead to large segmental bone defects [11]. The grafts which are necessary for the treatment of large scale bone defects often include autografts, allografts, xenografts and synthetic biomaterial scaffolds. However, because autografts are not sufficiently available, as well as allografts and xenografts have risks of disease transmission or adverse host immune responses [12], it is urgent to develop bone substitution materials by using synthetic biomaterial scaffolds. Biomedical scaffolds have been adopted to restore function and damage in human tissues and are often used in tissue engineering as a physical and biological support for tissue regeneration [9,13-17].

Development of scaffold materials for use in bone tissue engineering (BTE) that mimic both the structure and mechanical properties of the natural bone still remains unmet needs for scientists [9,13,15,18]. Scaffolds should provide a highly porous matrix with interconnected pores that allow the exchange of nutrients, oxygen and metabolic waste products [18]. The synthetic biomaterial scaffolds should provide an initial platform for cells to retain in the defects, support cell growth, and maintain phenotypes [16,19]. An ideal scaffold for BTE should allow cell adhesion, migration, proliferation and differentiation by carrying growth factors and other factors [8,20].

Recently, three-dimensional (3D) fabricated scaffolds have been considered one of the most promising materials in BTE because of their highly precise pore size, structure, interconnectivity, and mechanical properties [21-24]. Among these scaffolds, biodegradable polymer-based 3D scaffolds have attracted much attention in recent decades, such as poly(lactic acid) (PLA), poly(glycolic acid) (PGA), poly(ε-caprolactone) (PCL), or their random copolymer poly(lactic acid-co-glycolic acid) (PLGA) [25-31]. PCL has favorable characteristics of good biocompatibility and minimal inflammation and has been used in many FDA (Food and Drug Administration) approved implants, including drug delivery devices, adhesion barriers, suture materials, and *etc.* [32]*.* The PCL freeform fabrication has also been widely used in the fabrication of porous 3D scaffolds for BTE research [15,33].

HAp and collagen are both considered to be biomimetic and they are both components of natural bones. In BTE, HAp, a major inorganic component in natural bone, has been often used due to its high mechanical strength, osteoinductivity, and excellent biocompatibility [34,35]. HAp is believed to stimulate the formation, precipitation, and deposition of calcium phosphate from simulated body fluid, resulting in enhanced bone-matrix interface strength. The addition of HAp to polymer scaffolds has been shown to improve the bioactivity and mechanical properties and to potentially reduce adverse effects associated with the degradation of some synthetic polymer [13,36-39], such as PLLA (poly-l-lactide acid), PLGA, PMMA (Poly(methyl methacrylate)), *etc*. Likewise, it was reported that collagen coating promoted bone cell proliferation, improved cell adhesion and enhanced osteogenic cell differentiation [40,41]. More importantly, the initial adhesion of periosteum segments was greatly improved by collagen-coating, which thus facilitated cell outgrowth and handling efficiency on implantation [42]. At the same time, poly-lysine is often used to enhance cell binding by increasing electrostatic interaction between negatively-charged ions of the cell membrane and positively-charged surface ions of attachment factors on the culture surface. When adsorbed to the culture surface, it increases the number of positively-charged sites available for cell binding.

Our current study was designed to evaluate the osteoinduction capacity of 3D PCL scaffold for potential treatment of large segmental bone defects and further optimization by coating with poly-lysine, HAp, collagen or HAp/collagen to form favorable nanocomposites. To do so, bone-marrow derived stromal cells (BMSCs) were cultured in 3D PCL matrix. BMSCs exhibit multiple traits of a stem cell population. They have significant proliferative capacity and can be greatly expanded *in vitro* and induced to differentiate into multiple cell types. Under certain conditions, BMSCs are demonstrated to be osteogenic on biomaterials without addition of any other factors and have capacity to grow multiplicatively for a longer passage number than differentiated cells [43,44]. In summary, our goal is to design and optimize a precisely controlled 3D lattice that facilitates the attachment, survival, migration, proliferation, and differentiation of BMSCs and promotes a bone healing response. The results can be potentially utilized to the treatment of large segmental bone defects.

## Materials and methods

3D Biotech PCL scaffolds, Type I Collagen of calf skin, HAp, poly (L-Lysine), and β-actin antibody were purchased from Sigma-Aldrich, Runx2, and RANKL antibodies were purchased from Cell Signaling Technologies, Osteocalcin antibody was purchased from Santa Cruz, and Osterix and ALP antibodies were purchased from Abcam. All chemicals except indicated ones were purchased from Sinopharm Chemical Reagent Co (Shanghai, China). PCL scaffolds were immersed in 75% alcohol overnight before use.

### Surface with collagen coating

A collagen coating was completed by immersing the PCL scaffolds into a collagen solution (10 mg/mL and 30 mg/mL in 0.05 M acetic acid as indicated) overnight. Afterwards, the constructs were washed quickly three times with PBS (Phosphate Buffered Saline) and kept air-dried. Before cell seeding, the scaffolds were balanced with medium, and medium was changed three times.

### Surface with HAp coating

HAp powders with 15% of weight percentage relative to PCL was added into 2 ml THF/DMF (1/1 volume ratio). The mixture was sonicated for 1 hour and added to PCL scaffolds. The coating was completed by immersing the PCL scaffolds into HAp solution for 1 h. Afterwards, The scaffolds were immediately frozen in liquid nitrogen. Solvent extraction was performed in cold ethanol at −20°C; ethanol was changed three times. Afterwards, the constructs were washed quickly three times with PBS and kept air-dried. Then the scaffolds were subjected for medium balancing and cell seeding.

### Surface with HAp and collagen coating

PCL scaffolds were first coated with HAp as described, and then were coated with collagen as indicated. Afterwards, the constructs were washed quickly three times with PBS and kept air-dried. Medium balancing and cell seeding follow.

### Surface with poly-L-lysine coating

PCL scaffolds were immersed in 0.01% (W/V) poly-lysine water solution. After 3 hours, the solution was removed through aspiration and the scaffolds were rinsed thoroughly. PCL scaffolds were dried for two hours before the scaffolds were balanced with medium. And medium was changed three times before cell seeding.

### Isolation and expansion of BMSCs

The use of rats and all related procedures in this study were approved by Shanghai Jiao Tong University Affiliated Sixth’s People’s Hospital Animal Care and Use Committee. All rats were provided with sterilized food and water and housed in a barrier facility under a 12-hour light/dark cycle. As previously described, the bone-marrow samples were incubated in isolation medium (25 mM HEPES, 128.5 mM NaCl, 5.4 mM KCl, 5.5 mM D(+)-glucose, 51.8 mM D(+)-saccharose, 0.1% bovine serum albumin (Sigma, A6003)) overnight at 4°C, before centrifugation at 110*g for 15 min. The supernatant was discarded and the pellet was washed several times. The cell suspension was filtered through a 200 μm polyethylene terephthalate (PET) filter with subsequent centrifugation at 110*g for 15 min, after which the pelleted cells were resuspended in basal growth medium (MEM supplemented with 10% Fetal Bovine Serum (FBS; Gibco), 1% penicillin–streptomycin–neomycin antibiotic mixture (100×) (PSN; Gibco), 1 ng/mL fibroblast growth factor 2 (FGF-2; Sigma-Aldrich). The cells were seeded into T75 culture flasks at a density of 10^7^ per flask. 24 h later, the non-adherent cells were removed. The medium was changed twice a week, and subcultured prior to confluence until the seeding of the roughness gradients. For all experiments, cell passage one or two was used. 10^5^ cells/ml cells were seeded.

### Alkaline phosphatase activity

After 2 weeks of cell culture, cells were trypsinized from scaffolds and seeded onto the glass coverslips. The second day, media were removed and coverslips were washed with PBS for three times. Then cells were fixed with 4% PFA for 2 min and washed with PBS twice. After PBS was removed, TBST buffer (20 mM Tris–HCl pH 7.4, 0.15 M NaCl, 0.05% Tween-20) was added. After TBST was removed, ALP staining kit mixture (Yeasen Biotech, Shanghai, China) was prepared and added as instructed and incubated at room temperature for 4 hours in dark. Then the coverslips were washed with PBS once and kept in PBS until picture capture.

For quantification of ALP activity, after cells were fixed with 4% PFA for 2 min and washed with sterilized water twice, mixture of ALP detection kit ( Nanjing Jiancheng Bioengineering Institute, Nanjing, China) was added. Optical density was measured at 405 nm using a spectrometer.

### Cell viability and proliferation

Cells were seeded at 2.0*10^5^ cells/scaffold. One milliliter of medium was added into each well to immerse the scaffolds completely to be followed by culturing in an incubator at 37°C and 5% CO_2._ The growth medium used during first four days of culturing contained a 1:1 mixture of Ham’s F12 medium and Dulbecco’s modified Eagle’s medium-low glucose (Gibco, Life Technologies), supplemented with an antibiotic solution (100 U/ml penicillin and 0.1 mg/ml streptomycin, Life Technologies) and 10% fetal bovine serum (Gibco, Life Technologies). On day 4, the growth medium was replaced with the osteogenic differentiation medium, which was supplemented with 50 μg/ml ascorbic acid, 10 mmol β-glycerophosphate, and 10 nmol dexamethasone. The medium was changed every two days and the scaffolds were flipped during each medium change. At days 7, 14, 21, and 28, samples were harvested and characterized for cell differentiation analysis.

Cell proliferation was measured at 28 days post cell seeding using 3-(4, 5-Dimethylthiazol-2-yl)-2,5-diphenyltetrazolium bromide (MTT) assays. MTT is a tetrazole that can be metabolized and reduced to purple formazan in live cells. Assays were carried out in 12-well plates: final concentration of MTT is 0.5 mg/ml and incubated at 37°C for 4 h and culture medium was then removed. 0.5 ml of isopropanol was then added to each well to completely dissolve formazan crystals throughout the PCL scaffolds. Optical density was measured at 560 nm using a spectrometer. All data points were normalized to the viability of PCL scaffolds without coatings.

### Western blotting

Cells were lysed in MPER™ (Mammalian Protein Extraction Reagent, Thermo Scientific, Rockford, IL), which was supplemented with cOmplete Protease Inhibitor Cocktail tablets and PhosSTOP Phosphatase Inhibitor Cocktail tablets (Roche, Indianapolis, IN). Protein concentrations of lysates were determined using the BCA assay (Thermo Scientific, Rockford, IL) and proteins were resolved on SDS-PAGE gels and transferred to PVDF membrane (EMD Millipore, Billerica, MA) using standard procedures. Membranes were blocked with SuperBlock™ T20 (TBS) Blocking Buffer (Thermo Scientific, Rockford, IL) and incubated overnight at 4°C in primary antibodies of Runx2 (Cell Signaling, #8486, 1:1000), ALP (Abcam, #ab67228, 1:1000), RANKL (Cell Signaling, #4816, 1:1000), Osteocalcin (Santa Cruz, #sc-30044, 1:500), Osterix (Abcam, #ab22552, 1:500), and β-actin (Sigma-Aldrich, #A1978, 1:5000). Membranes were then incubated with either anti-mouse (Cell Signaling, #7076, 1:2000) or anti-rabbit IgG (Cell Signaling, #7074, 1:2000), HRP-linked secondary antibodies and Chemiluminescented by Pierce™ ECL Western Blotting Substrate (Thermo Scientific, Rockford, IL). Quantification of signals on Western blots was done using National Institutes of Health (NIH) ImageJ Imaging and Processing Analysis Software with signaling intensity normalized to β-actin.

### RNA isolation, reverse transcription, and quantitative real-time PCR (qRT-PCR)

Primers used in the present study are listed in Table 1. All primers were synthesized by Sangon Biotech (Shanghai, China). Cells were first typsinized and harvested from the scaffolds and cell pellets were stored at −80°C. Then RNeasy kit (Qiagen) was used for total RNA extraction and isolation following manufacture’s instructions. Briefly, frozen cell pellets were thawed slightly and lysed and homogenized. Ethanol was added and the samples were transferred to to a RNeasy spin column. The total RNA was then washed and eluted. Reverse transcription was done with RevertAid First Strand cDNA Synthesis Kit (Thermo Scientific, Rockford, IL) following manufacture’s instructions. Briefly, cDNA was synthesized using the ReveriAid M-MuLV reverse transcriptase with the primers, RNase inhibitors, dNTP mix from the kit. qRT-PCR were performed in triplicate by Bio-Rad real-time PCR system with iQ5 SYBR Green SuperMix (Bio-Rad) for 40 cycles at 95°C for 30 s, 53–60°C annealing temperature for 30 s, and 72°C for 60 s. Melting curve analysis was performed using Biorad 7300 Syntem SDS software.

**Table 1:** ***Rattus norvegicus*** **primers used in the qRT-PCR experiments**

**Gene name**	**Accession number**	**Primer (forward)**	**Primer (reverse)**
Runt-related transcription factor 2 (Runx2)	XM_001066909	5′-TAACGGTCTTCACAAATCCTC-3′	5′-GGCGGTCAGAGAACAAACTA-3′
Osterix	AY177399	5′-GCCTACTTACCCGTCTGA-3′	5′-CTCCAGTTGCCCACTATT-3′
Alkaline phosphatase (ALP)	NM_013059	5′-GCAGGATCGGAACGTCAAT-3′	5′-ATGAGTTGGTAAGGCAGGGTC-3′
Osteocalcin	J04500	5′-CACAGGGAGGTGTGTGAG-3′	5′-TGTGCCGTCCATACTTTC-3′
Receptor activator of nuclear factor- κB ligand (RANKL)	NM_057149	5′-TCGCTCTGTTCCTGTACT-3′	5′-AGTGCTTCTGTGTCTTCG-3′
β-actin	NM_031144	5′-CAGAGCAAGAGAGGCATCCT-3′	5′-GTCATCTTTTCACGGTTGGC-3′

### Scanning Electron Microscope (SEM) analysis

Field Emission Scanning Electron Microscope S-4800 (Hitachi, Japan) was used to characterize the scaffolds, as well as the morphology and behavior of the cells grown in the scaffolds. Prior to imaging, the cells were fixed with 2.5% glutaraldehyde and samples were dehydrated in a graded ethanol series and sputter-coated with gold for 15-20s. All samples were analyzed at 15 kV.

### Statistical analysis

Statistically significant differences between different groups were determined by two-way ANOVA, followed by Bonferroni post tests with P < 0.05 considered significant (*, P < 0.05; **, P < 0.01; ***, P < 0.001) using GraphPad Prism version 5.00 (San Diego, California) unless otherwise specified.

## Results

### Morphology of uncoated and coated PCL scaffolds

**Figure 1:**
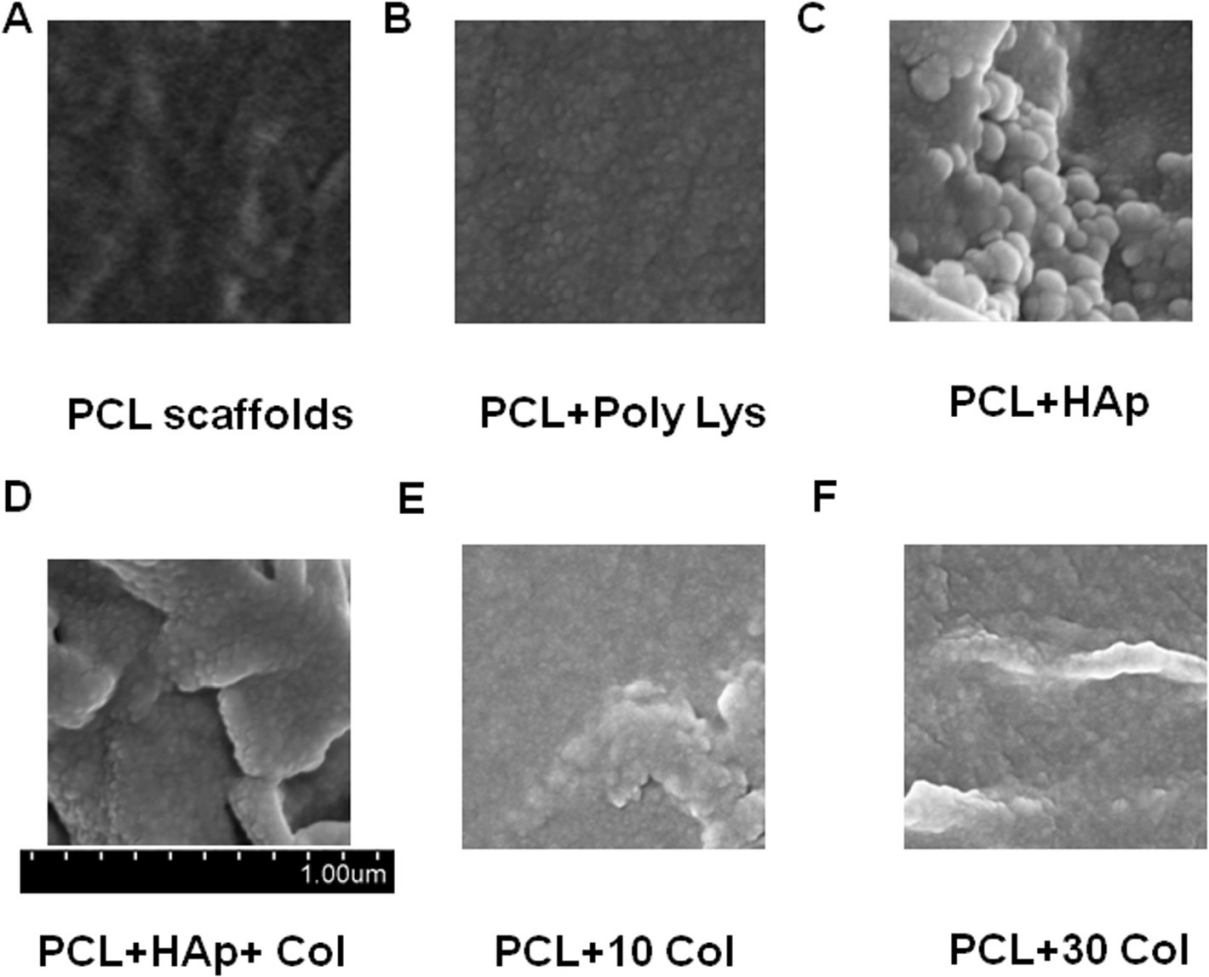
Representative SEM micrographs of PCL scaffolds after coating. The micrographs show the details of scaffolds after coating. **A**, PCL scaffolds only; **B**, PCL scaffolds with poly-lysine coating; **C**, PCL scaffolds with HAp; **D**, PCL scaffolds with HAp and 30 mg/ml collagen coating; **E**, PCL scaffolds with 10 mg/ml collagen coating; **F**, PCL scaffolds with 30 mg/ml collagen coating.

Since the potential advantages of HAp, collagen, and poly-lysine coatings have been discussed earlier, therefore we tried to coat PCL scaffolds with poly-lysine, HAp, collagen or both HAp and collagen and evaluated the morphology using SEM. Under SEM, uncoated PCL scaffold showed a relatively smooth surface (Figure 1A). However, it could be observed that the coated PCL scaffolds had surface roughness and coating materials were successfully and uniformly deposited on the surface of PCL scaffolds (Figure 1B-F). Of note, compared to other PCL scaffolds, the HAp-Col and HAp PCL scaffold presented microstructure with more nanoparticles and roughness on the surface, better mimicking the micro/nano-structure of the natural bone and also increasing the accessible surface for cells.

### Adherence of BMSCs to the uncoated and coated PCL scaffolds

**Figure 2:**
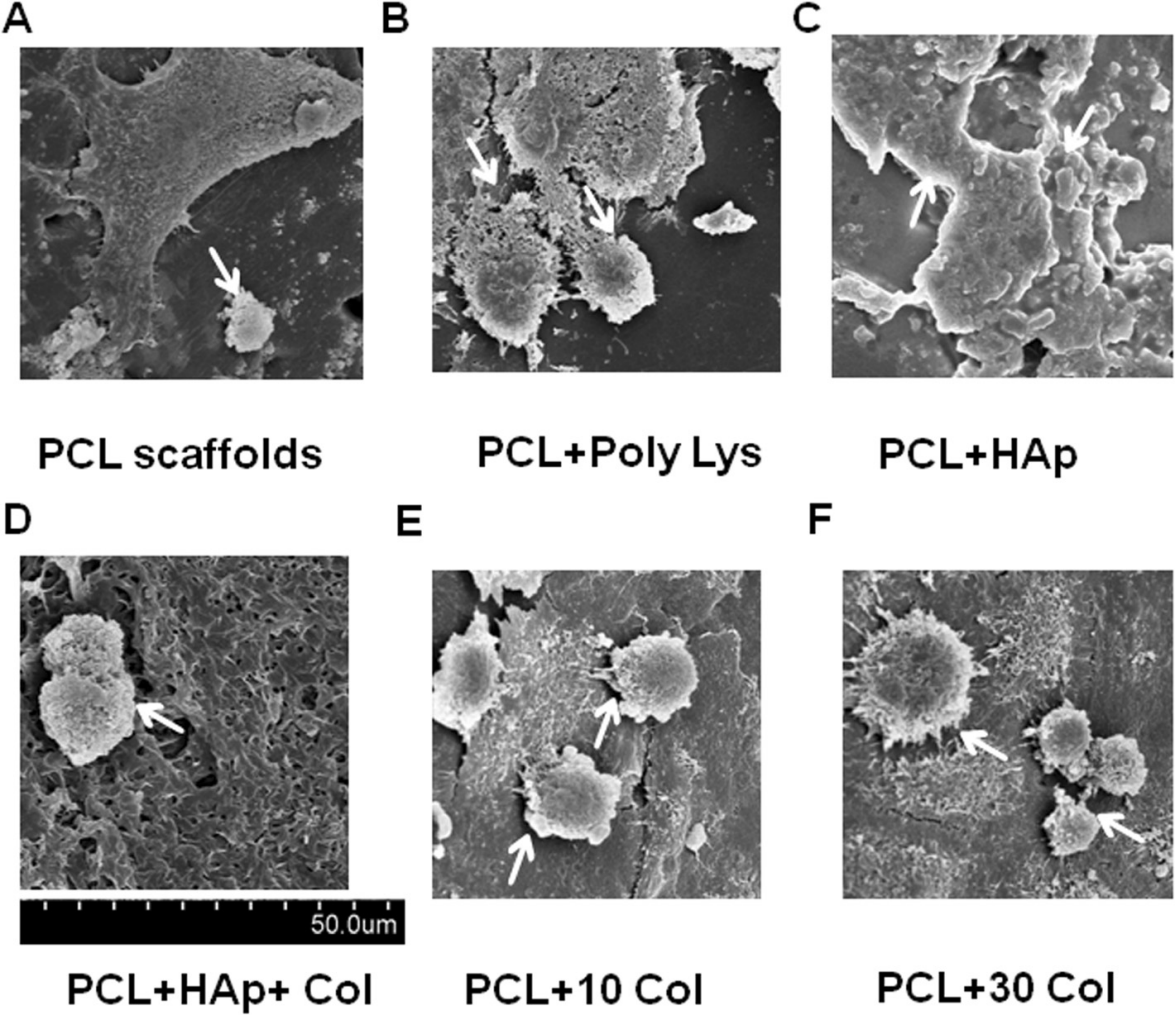
Representative SEM micrographs of PCL scaffolds after coating and 28 days of culturing. The micrographs show the proliferation and spreading of BMSCs (white arrows) after 28 days in culture. **A**, PCL scaffolds only; **B**, PCL scaffolds with poly-lysine coating; **C**, PCL scaffolds with HAp; **D**, PCL scaffolds with HAp and 30 mg/ml collagen coating; **E**, PCL scaffolds with 10 mg/ml collagen coating; **F**, PCL scaffolds with 30 mg/ml collagen coating.

BMSCs have been previously shown having great potential for bone regeneration. Under certain conditions, they could be induced to differentiate into osteoblasts. Moreover, several scaffolds have been demonstrated to promote the growth and differentiation of BMSCs. Therefore, in order to evaluate cytocompatibility of these uncoated and coated PCL scaffolds, BMSCs were seeded into the scaffolds. After 28 days, SEM observation revealed that cells were well attached to the poly-lysine, HAp, HAp/collagen and collagen coated scaffold surface and grew in colonies on the surface (Figure 2). These results indicated that all these coated scaffolds possessed favorable cytocompatibility and provided suitable circumstances for cell growth.

### Osteogenic differentiation of BMSCs growing on uncoated and coated PCL scaffolds

**Figure 3:**
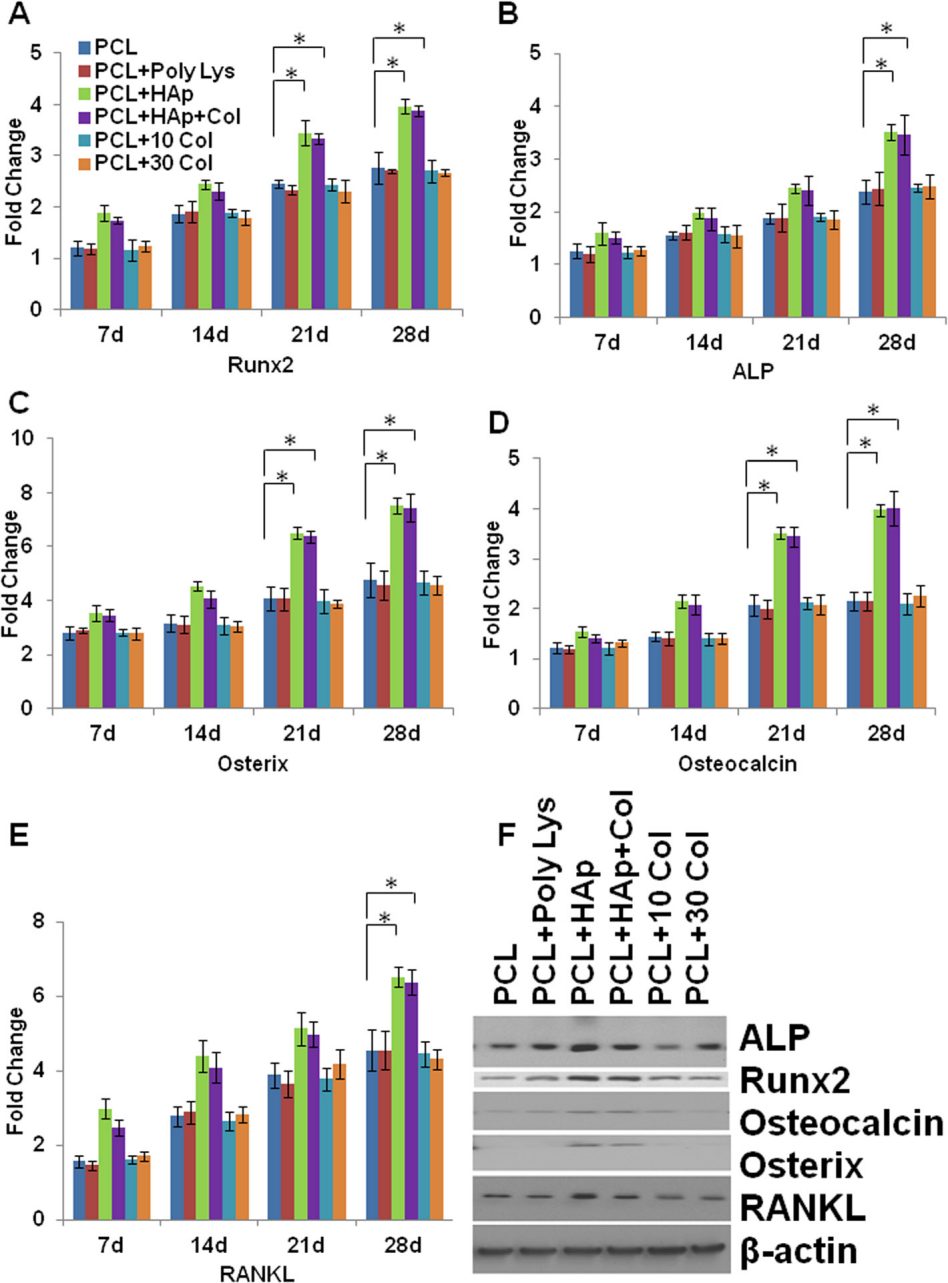
Osteogenic differentiation of BMSCs growing on scaffolds. The mRNA expression of bone tissue specific markers, including Runx2 **(A)**, ALP **(B)**, Osterix **(C)**, Osteocalcin **(D)**, RANKL **(E)** were evaluated during the in vitro culturing period of up to 28 days to assess osteogenic differentiation of BMSCs by RT-qPCR at different time points. All data were normalized to the mRNA expression of the corresponding marker at day 0 (cell seeding day). Data indicate the mean relative values calculated from six independent experiments (±S.E.). Statistically significant differences with P < 0.05 were considered significant (*P < 0.05). **F**, shows a representative protein expression (as indicated) of cell lysates cultured on PCL scaffolds only, PCL scaffolds with poly-lysine coating, PCL scaffolds with HAp, PCL scaffolds with HAp and 30 mg/ml collagen coating, PCL scaffolds with 10 mg/ml collagen coating, PCL scaffolds with 30 mg/ml collagen coating after 28 days of cell culturing.

Recruitment, proliferation and differentiation of BMSCs into mature osteoblasts are the major processes of osteogenesis and remain challenging in the context of bone tissue engineering. The process of osteogenesis can be characterized by identifying temporally regulated bone formation markers. In BMSCs, runt-related transcription factor 2 (Runx2), ALP, osterix and osteocalcin are the main determinants of osteogenesis [45-48]. Runx2 and osterix are the key transcription factors initiating and regulating the early osteogenesis and late mineralization of bone. The production of ALP and osteocalcin is an early biomarker of osteogenesis. RANKL is a ligand for osteoprotegerin and functions as a key factor for osteoclast differentiation and activation. Thus, these key osteogenesis-related markers were determined to evaluate the differentiating capacity of BMSCs in the scaffolds. Gene expression analysis in the BMSCs seeded on the different scaffolds showed a time-dependent upregulation of osteogenesis-related markers including RUNX2, ALP, osterix, osteocalcin and RANKL. In particular, mRNA levels of RUNX2, osterix, and osteocalcin were significantly higher in BMSCs seeded in HAp PCL and HAp-Col PCL scaffolds than those seeded in uncoated PCL scaffolds at days 21 and 28. In addition, mRNA levels of ALP and RANKL were also significantly higher in BMSCs seeded in HAp PCL and HAp-Col PCL scaffolds than those seeded in uncoated PCL scaffolds at days 28 (Figure 3A-E). At the same time, mRNA expression levels of the above markers were not significantly different in collagen or poly-lysine coated PCL scaffolds from uncoated PCL scaffolds (Figure 3A-E). Additionally, protein expression levels of RUNX2, ALP, RANKL, Osterix and Osteocalcin of BMSCs were also much higher in HAp-Col PCL and HAp PCL scaffolds than in uncoated or poly-lysine and collagen coated scaffolds (Figure 3F), indicating that both HAp-Col PCL and HAp PCL scaffolds have favorable osteoinductive potential.

### ALP activity and cell proliferation

**Figure 4:**
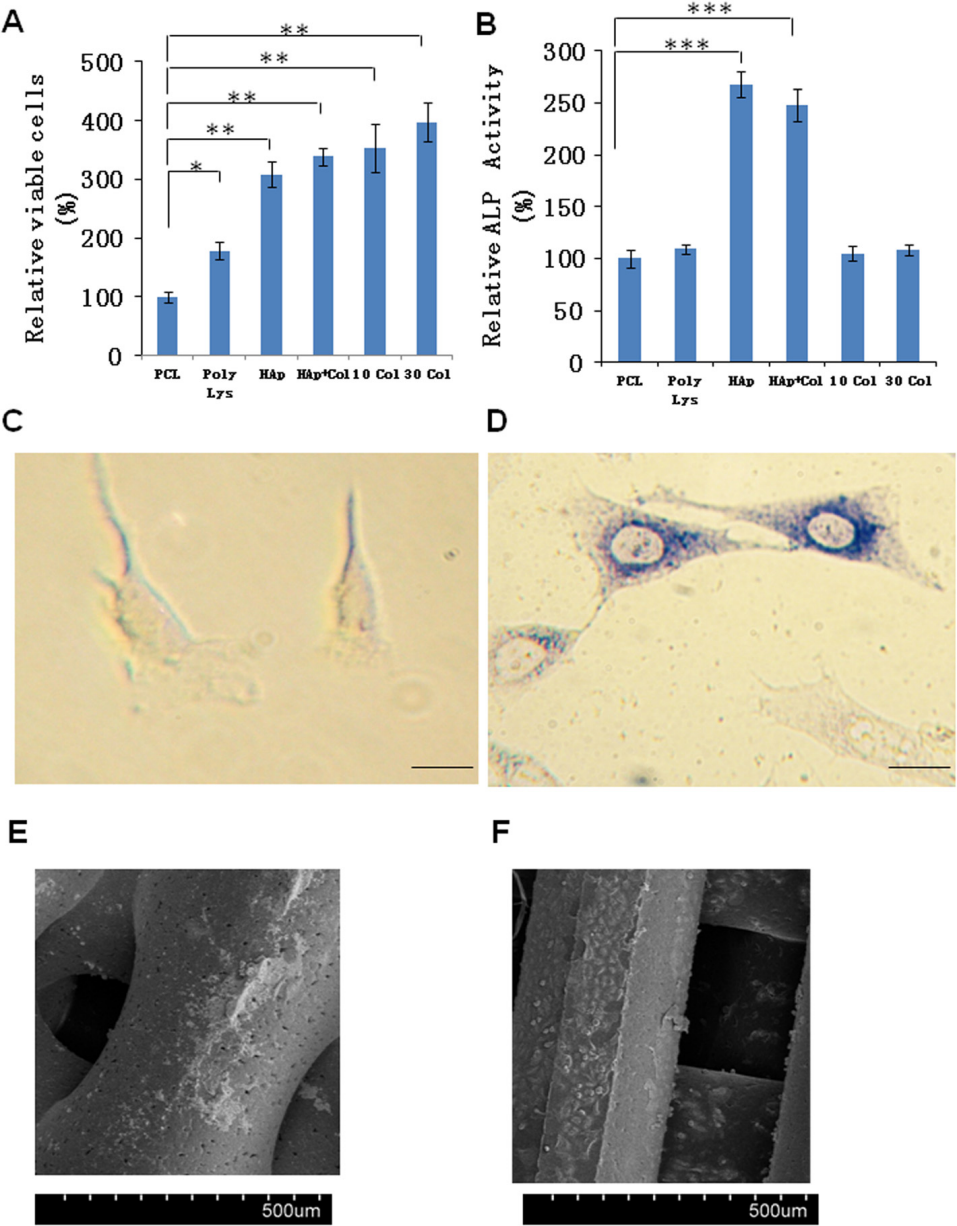
Cell-scaffold interactions on PCL scaffolds. BMSCs were cultured for 4 weeks *in vitro*. **A**, cell proliferation of BMSCs on PCL scaffolds with or without coatings as indicated. Cell proliferation were determined via MTT assays. Viability of cells were normalized to the viability on PCL scaffolds only. **B**, alkaline phosphates (ALP) activity after 4 weeks of BMSC culturing. Data were normalized to the ALP activity on PCL scaffolds only. Data indicate the mean relative values calculated from six independent experiments (±S.E.). Statistically significant differences with P < 0.05 were considered significant (*P < 0.05; **P < 0.01; ***P < 0.001). **C**, representative BMSC image before culturing on PCL scaffolds with HAp and 30 mg/ml collagen coating. Scale bar, 10 μm. **D**, representative cell image after culturing on PCL scaffolds with HAp and 30 mg/ml collagen coating. The cells have differentiated into osteoblastic cells and were stained with ALP staining kit. SEM micrographs of PCL scaffolds after coating and 4 weeks of culturing. Scale bar, 10 μm. **E**, PCL scaffolds only. **F**, PCL scaffolds with HAp and 30 mg/ml collagen coating.

Next, cell proliferation among groups was determined quantitatively using MTT assay. Interestingly, results at 28 days showed that collagen-coating significantly promoted cell proliferation. As shown in Figure 4A, all coatings significantly increased cell proliferation at days 28. Specifically, collagen (10 Col, 30 Col), HAp-Col, and HAp coated PCL scaffolds had higher cell proliferation compared to viable cells in poly-lysine coated or uncoated PCL scaffolds, suggesting that the presence of collagen and HAp additionally stimulated BMSC proliferation in coated scaffolds. As we know, collagen and HAp are both components of natural bones and these components may help stimulate BMSC proliferation and provide additional surface for cell attachment according to their SEM results (Figures 1 and 2).

Osteogenic differentiation of BMSC could show ALP activity which can be assessed by ALP activity assays. BMSCs in HAp-Col PCL and HAp PCL scaffolds had remarkably higher ALP activity than those in collagen-only coated or uncoated PCL scaffolds (Figure 4B), indicating HAp and HAp-Col may help osteogenic differentiation and stimulate osteoblast formation. Taken together, the scaffolds with both HAp and collagen coatings have the advantages of both components, and provide the best conditions for both osteogenesis and proliferation of BMSCs in 3D PCL scaffolds. Before cultured in the scaffolds, BMSC showed negative stain of ALP activity (Figure 4C). However, after 28 days of culture in HAp-Col PCL scaffolds, cells abundantly expressed ALP activity and showed spread-out phenotype and were connected with each other (Figure 4D). These morphology changes of BMSCs before and after four-week of 3D culture again confirmed that BMSCs successfully differentiated into osteoblast in HAp/collagen coated PCL scaffolds. Further SEM micrograph confirmed that in contrast to cells in uncoated PCL scaffold, BMSCs in the HAp-Col PCL scaffold were well attached and spread throughout the scaffold (Figure 4E-F). In summary, our data strongly suggested that the HAp-Col PCL scaffold, mimicking the organic matrix and inorganic phase of native bone, greatly promoted BMSC attachment, proliferation and differentiation.

## Discussion

Currently biodegradable polymer/bioceramic composite was viewed as a suitable scaffold material in BTE because the addition of a polymer phase to a porous ceramic scaffold increases the fracture toughness of the composite and allows the functionalization of the surface to induce enhanced bioactivity. In this study, PCL was chosen as the polymer layer as it is known to be biodegradable with good biocompatibility and non-inflammation, and has the additional advantage of FDA approval for implant applications. HAp, was chosen as the bioceramic layer, for the evidence that it is bioactive. In particular, HAp material possesses excellent biocompatibility in contact with bones, teeth, skin, and muscles. Furthermore, HAp promotes faster bone regeneration as well as direct bonding to regenerated bones without intermediate connective tissues. In consistent with other groups, our data clearly demonstrated that HAp-coating produced nano-featured surface in Figure 1 [49,50]. Additionally, HAp-coating subsequently facilitated BMSC homing and osteogenesis in vitro in Figure 2 and Figure 3, respectively. As shown in Figure 2C, cell clustered much more densely on HAp-coated PCL scaffolds than the scaffolds alone. Furthermore, in Figure 3 the mRNA expression of many biomarkers in scafffolds coated with HAp were significantly greater than PCL scaffolds alone at day 21 and 28. All these results showed HAp-coating is favorable for osteoinduction and proliferation of BMSCs. Furthermore, in our study, the method of HAp coating is simple to perform.

To the best of our knowledge, collagen is seldomly coated together with HAp to PCL scaffolds. In the present study, although BMSCs showed similar osteogenesis in HAp-only coated PCL scaffolds and HAp-col-coated PCL scaffolds (as shown in Figure 3), combination coating of HAp and collagen significantly enhanced BMSC survival and proliferation (as shown in Figure 4A). As shown in Figure 4A, all coatings significantly increased cell survival, especially collagen coatings. As we all know, for native bone tissue, collagen plays a dominant role in maintaining the biological and structural integrity of ECM (extracellular matrix) [51]. Studies have shown that cells bind collagen and other ECM proteins using adhesive transmembrane molecules such as integrins, thus increasing the efficiency of cell adhesion [52]. In addition, collagen increases tissue vascularization and decreases the inflammatory response and macrophage and osteoclast activity. Though HAp coatings are valuable due to their surface chemistry and have been shown to enhance osteoblast proliferation and differentiation, they often suffer from high brittleness. Collagen provides the necessary environment for cell attachment and the ductile properties of collagen also help to increase the poor fracture toughness of HAp [52]. In fact, our results have confirmed that the coating with both collagen and HAp simultaneously enhanced not only BMSC proliferation but also osteogenesis based on the advantages of the characterics of both components of natural bones. It is known that osseous tissue is a relatively hard and lightweight composite material which is mostly made up of a composite incorporating the mineral calcium phosphate in the chemical arrangement termed calcium hydroxylapatite, which gives bones their rigidity, and collagen protein which improves fracture resistance. Therefore, our coating of both HAp and collagen on PCL scaffolds mimicks the biophysical and biochemical properties of natural bone tissue very well, and will potentially become an efficient strategy to build tissue-engineered scaffolds. Additionally, Figure 1 and 2 showed that the PCL-HAp-collagen scaffolds had porous structures with collagen nanofibers and HAp nanoparticles on the surface. These features of such PCL scaffolds are again favorable for cell attachment and osteogenesis since the main constituents of bone are nanometers in diameter and native bone cells interact with nano-scale proteins and minerals. The pore sizes are about 570 μm, which is appropriate for bone regenerative cell migration and infiltration.

In our results, though poly-lysine and collagen coatings increased attachment and proliferation of cells, they cannot enhance BMSC differentiation as demostrated by lower expression of osteogenesis-related markers compared to HAp and HAp-Col coated scaffolds in Figure 3A-F and Figure 4A-B. These poly-lysine and collagen coatings seemed to be lack of the ability to accelerate osteogenesis BMSC differentiation. PCL scaffolds need inorganic phase of native bone, a necessary component, to mimick the complete matrix for promoting BMSC differentiation. These results further confirmed that HAp plays a pivotal role in maintaining biocompatibility and osteoinductivity of the scaffolds in the context of BTE.

To the best of our knowledge, the interaction between BMSCs and PCL scaffolds coated with both HAp and collagen haven’t been studied throughly by any other groups yet. The incorporation of BMSCs into BTE biomaterials is an accepted strategy for accelerated bone formation and osteointegration during bone defect repair and regeneration [9]. This present study introduces an easy method to immobilize BMSCs on the 3D porous PCL scaffolds and provides further evidence that modification of 3D PCL scaffolds with both HAp and collagen elicits specific cellular responses and improves the final cell-biomaterial interaction. This finding suggests that the co-coating of both HAp and collagen promotes regeneration and that the system has potential in BTE.

## Conclusions

This study demonstrates that HAp-collagen coated PCL scaffolds possess appropriate nano-structures, surface roughness, and nutrients, offering a favorable environment for osteogenesis. The co-coating of collagen and HAp in the PCL scaffolds promotes BMSC proliferation and differetiation which play essential roles in the physiological process of ostogenesis. HAp-collagen coated PCL scaffolds take the advantages of all materials, providing promising potential for bone tissue engineering applications.
